# New chalcone derivatives as potential antimicrobial and antioxidant agent

**DOI:** 10.1038/s41598-021-01292-5

**Published:** 2021-11-05

**Authors:** Emelda N. Okolo, David I. Ugwu, Benjamin E. Ezema, Joseph C. Ndefo, Florence U. Eze, Chidimma G. Ezema, James A. Ezugwu, Oguejiofo T. Ujam

**Affiliations:** 1 Department of Pure and Industrial Chemistry, University of Nigeria, Nsukka, Nigeria; 2 Department of Science Laboratory Technology, University of Nigeria, Nsukka, Nigeria; 3 National Center for Energy Research and Development, University of Nigeria, Nsukka, Nigeria

**Keywords:** Drug discovery, Chemistry

## Abstract

Seven chalcone derivatives were synthesized by the Claisen-Schmidt condensation. The structures of the compounds were confirmed by spectral data (Ultraviolet/visible, infrared, nuclear magnetic resonance and mass spectroscopy). The compounds were tested for their in silico and in vitro antimicrobial and antioxidant activities. The molecular docking assessments showed that all the compounds exhibited good binding affinity with the target microorganism proteins but, compounds **6e** and **6g** showed better binding affinity compared with the standards. The antimicrobial test revealed that all the compounds screened were active against S*taphylococcus aureus and Bacillus subtilis* and had minimum inhibitory concentrations (MIC) between 0.4 and 0.6 mg/mL. Compounds **6a**, **6c** and **6d** had moderate activities on *Salmonella typhi.* Compounds **6b** and **6c** had moderate activity on *Escherichia coli.* Compound **6c** had moderate activity on Aspergillus *niger* while compounds **6a** and **6e** had poor activity. All the compounds except compound **6e** had no inhibition against *Pseudomonas aeruginosa.* The *in-vitro* antioxidant activity was assessed using ethylenediaminetetraacetate (EDTA) as the standard. Compounds **6c**, **6e** and **6g** gave excellent inhibitory activity better than the standard. Compound **6a** gave good activity at 500 μg/mL and 1000 μg/mL concentrations but, below the standard at 250 μg/mL and no inhibition at 125 μg/mL. Compound **6d** had good inhibition at 500 μg/mL and 1000 μg/mL but, no inhibition at 125 μg/mL and 250 μg/mL. Compound **6b** was found to be inactive in all the concentrations. Absorption, distribution, metabolism and excretion properties of the compounds were assessed using SwissADME. The results of lead likeness showed that compound 6e is a lead-like molecule.

## Introduction

The success recorded in the treatment of infectious diseases is consistently challenged by continues report of bacterial resistance. The mechanism of resistance is usually encoded genetically and as such can be transferable^1^. This wide increase in resistance mechanism negatively affects the therapeutic efficacy of a whole class of drugs^2^.

Oxidative stress is implicated in many human diseases^3^. Elevation of superoxide anions, hydrogen peroxide, hydroxyl, nitric oxide and peroxynitrite causes damage to many cellular macromolecules including DNA^4,5^. These damages often leads to diabetes, atherosclerosis, myocardial infarction, damage may result into many diseases including diabetes mellitus, atherosclerosis, mycocardial infarction, arthritis, anemia, asthma, inflammation and many more^6^. However, human cells uses superoxide dismutase, catalase, glutathione reductase, ascorbic acid and other enzymatic and non-enzymatic mechanism to stop the production of free radicals^7^. The protective role of these enzymes are often times disrupted during pathological processes thereby necessitating the use of antioxidant supplements or drugs. The reported human and animal toxicity to many antioxidants like butylated hydroxyanisole (BHA), butylated hydroxytoluene (BHT) and acidity of ascorbic acid prompted the search for new antioxidants^8^.

Chalcones are structural derivatives of 1,3-diphenylprop-2-en-1-one. They are ubiquitous in natural products and belong to the family of flavonoids examples licochalcone A (**1)** licochalcone D (**2)** and morachalcone A (**3**)^9,10^. They have been reportedly used as anticancer^11,12^, antidiabetics^13^, antioxidants^14^, antimalarial^15,16^, antitubercular^17,18^, antiviral^19^, anti-inflammatory^20,21^, antibacterial^22,23^ agents etc. Furthermore, chalcones are industrially used as light stabilizing agent^24^, sweetening agent^25^, analytical reagent in amperometry^26^, spectrometric reagent^27^ and synthetic reagent for the synthesis of pharmacologically active heterocyclic compounds^28–30^.
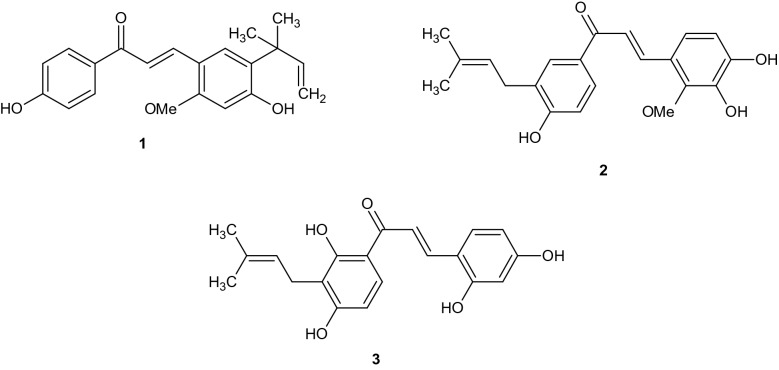


The immediate response of antigen is the Reactive-Oxygen Species (ROS) appearance in the body during microbial invaders. Free oxygen radicals are very toxic to pathogen and are used as agent to prevent attack of tissues by microorganism^31^.

Oxidative stress is seen as the main factor concerned with the development of chronic diseases and it happens when oxygen radical production and levels are higher than those of the antioxidant^32^. The importance of ROS for immune function could be used by the microbes to reduce defense mechanism of the host to survive. One of the important characteristic of plants accountable for antioxidant activity is the presence of derivatives of phenol and the power to hunt free radicals which can act as immunity against the harmful action of ROS. So antagonizing the production of ROS and free radical by addition of antioxidants can play important function in averting these free radical related diseases^33^.

Chalcones have preventive effects against many microorganisms. The antimicrobial effects are due to reactions between these compounds and the cell membrane of the target microorganism, their ability to attach with outer cell, absorbable proteins and the cell walls^34^. It is therefore possible to think that chalcones could inhibit the microbes through their antioxidant properties.

Glucosamine-6-phosphate synthase is responsible for the metabolism of hexosamine, an important process in the biosynthesis of amino sugars needed for cell growth and development. UDP-3‐O‐((*R*)‐3‐hydroxymyristoyl)‐*N*‐acetylglucosamine deacetylase is involved in the biosynthesis of lipid A, a phosphorylated glycolipid that anchors the lipopolysaccharide to the outer membrane of the cell^35^. DNA gyrase is involved in the control of topological transition of DNA, thereby promoting replication and transcription^36^. Urate oxidase catalyse the oxidation of uric acid to allantoin and the inhibition of this enzyme leads to accumulation of toxic uric acid in the microorganisms. Dihydrofolate reductase is an important enzyme in the conversion of pteridine to folic acid required by all cells for growth and development. Given the roles of these enzymes in the growth and development of organisms, their successful inhibition have been characterized as therapeutic target for drug development research.

This work was designed based on the reported pharmacological application of chalcones and the need for an antimicrobial host to have some antioxidant abilities to attack the reactive oxygen species produced by microorganism.

We herein report the synthesis of some new chalcones with good antimicrobial and antioxidant activities.

## Materials and methods

The chemicals used for experimental were of analytical grade purchased from Sigma-Aldrich and used it without purification. Melting points were determined using Fischer John's melting point apparatus and were not corrected. UV–Visible spectra were recorded on UV5800PC series spectrophotometer using matched 1 cm quartz cells. The IR spectra were recorded on Buck Scientific m910 FTIR U S A using KBr discs. Jeol 400 MHz was used for NMR whereas Waters Q-TOF premier HAB213 was used for Mass spectroscopy.

## Experimental

### Synthesis of chalcone derivatives

Acetophenone (**4)** (0.01 mol) and substituted benzaldehyde (**5**) (0L.01 mol) were mixed in a round bottom flask. Ethanol (30 mL) was annexed and then 40% potassium hydroxide (15 mL). The mixture was stirred for 30 min at room temperature, then left to stand for 24 h. The mixture was poured into a beaker containing crushed ice to quench the reaction and then neutralized with 10% HCl. The precipitates formed were filtered, washed with distilled water and dried. They were then recrystallized from absolute ethanol to obtain the desired products (**6–12**).

#### (2*E*)-3-(3-nitrophenyl)-1-phenylprop-2-en-1-one (6)

Yield = 87%, m.p = 107–109 °C, UV–visible (EtOH) λmax(nm) log(ε): 252 (2.852), 314 (2.6114), 360(2.5375), IR(KBr)v; 3801 (2v, (C=O), s-trans), 3695 (2v, (C=O), s-cis), 3178 (C-H stretch), 1850 (v(C=O) stretch), 1601 (C=C stretch), 1393 (C-H bending), 1184 (C-N stretch). ^1^HNMR (400 MHz, CDCl_3_ δ): 8.505–8.496 (m, 1H, ArH), 8.258–8.234 (m, 2H, ArH), 8.111–8.029 (m, 2H, ArH), 7.91 (d, J = 7.2 Hz, 1H,ArH), 7.843–7.803 (m, 1H, ArH),7.634–7.586 (m, 2H, ArH), 7.524–7.450 (m, 2H, ArH), 7.249–7.203 (m,1H, ArH) ^13^CNMR(400 MHz, CDCl_3_ δ): 189.825 (C=O of ketone),148.808, 141.771, 137.643, 136.727, 134.458, 133.438, 130.158, 128.909, 128.709, 128.575, 124.771, 124.685, 122.444, (13 aromatic carbon). ESI-MS: (m/z) M^+^ 253.1354.

#### (2*E*)-3-(4-chlorophenyl)-1-phenylprop-2-en-1-one (7)

Yield = 82%, mp: 204–206 °C, UV–visible (EtOH) λmax (nm) log (ε), 248 (2.6356), 290 (2.4651), 636 (0.2022). IR (KBr) v: 3806 (2v, (C=O), S-trans), 3700 (2v, (C=O), s-cis), 3219 (C-H stretch), 1817 (v(C=O) stretch, 1616 (C=C stretch), 821 (C–Cl stretch). ^1^HN MR (400 MHz, DMSO, δ): 8.304 (m, 1H, ArH), 8.171–8.149 (m, 2H, ArH), 7.997–7.982, 7.99 (d, 5.96 Hz, 1H, ArH), 7.714–7.691, 7.70 (d, 9, 2 Hz, 2H, ArH), 7.113–7.052 (m,5H, ArH), 6.867 (m, 1H, ArH), 6.383–6.364 (m,1H, ArH) ^13^CNMR (400 MHz, DMSO, δ): 195.584 (C=O), 153.472, 149.924, 149.552, 147.224, 137.877, 131.057, 129.416, 128.720, 125.820, 124.762, 124.504, 113.411. ESI-MS: (m/z) M^+^ 242.0495.

#### (2*E*)-3-(3, 4-dimethoxyphenyl)-1-phenylprop-2-en-1-one (8)

Yield 76% mp. 72–74 °C UV–visible (EtOH) λmax (nm) log (ε): 246 (2.4338), 294 (2.2628), 404 (2.4761). IR (KBr)v: 3796 (2v, (C=O), s-trans), 3488 (2v, (C=O), s-cis), 3058 (C-H stretch), 1870 (v(C=O) stretch), 1616 (C=C stretch), 1430 (C-H bending), ^1^HNMR (400 MHz, CDCl_3_, δ): 8.011–7.925 (m, 1H, ArH), 7.786–7.687 (m, 1H, ArH), 7.588–7.360 (m, 3H, ArH), 7.301–7.151 (m, 2H, ArH), 7.125–6.983 (m, 1H, ArH), 6.902–6.721 (m, 1H, ArH), 6.681–6.551 (m, 1H, ArH), 3.942 (s, 3H, –CH_3_), 3.806 (s, 3H, –CH_3_) ^13^CNMR (400 MHz, CDCl_3_, δ): 198.888 C=O, 145.162, 137.038, 133.177, 132.678, 128.683, 128.291, 128.118, 127.783, 127.448, 127.128, 124.880, 123.290, 120.176, 119.084, 111.219, 110.931, 56.055, 45.201. ESI-MS: (m/z) M^+^ 268.0634.

#### (2*E*)-3-(3-phenoxyphenyl)-1-phenylprop-2-en-1-one (9)

Yield, 88%, (Liquid), IR (KBr: cm^1^) 3848 (2v, (C=O), s-trans), 3446 (2v, (C==O), s-cis), 3050 (C–H stretch), 1878 (v(C=O) stretch), 1632 (C=C stretch), 1317 (C=C bending), 765 (phenyl bending). ^1^HNMR (400 MHz, CDCl_3_, δ): 8.002–7.980 (m, 1H. ArH), 7.940–7.904 (M, 1H, Ar H), 7.760–7.698 (m, 1H, ArH), 7.597–7.417 (m, 3H ArH), 7.383–7.344 (m, 2H, ArH), 7.298–7.121 (m, 3H, ArH), 7.084–7.008 (m, 2H, ArH), 6.971–6.910 (m, 1H, ArH,), 6.856–6.779 (m, 1H, ArH), 6.734–6.716 (m, 1H, ArH). ^13^CNMR (400 MHz CDCl_3_, δ): δ198.514 (C=O), 190.534(C-O),157.932, 157.329, 157.185, 156.840, 145.947, 144.184, 133.225, 132.975, 130.370, 130.015, 129.776, 128.751, 128.224, 123.788, 122.839, 120.876, 119.141, 118.806, 118.222. ESI–MS: m/z, M-H, 299.0314.

#### (2*E*)-3-(3-hydroxy-4-methoxyphenyl)-1-phenylprop-2-en-1-one (10)

Yield, 79%, mp: 110–112 °C, UV–visible (EtOH) λmax (nm) log (ε): 246 (2.308), 294 (2.13860, 422 (2.4702). IR (KBr cm^1^): 3811 (2v, (C=O), s-trans), 3662 (2v, (C=O), s-cis), 3167 (OH stretch), 3011(C-H stretch), 1838 (v(C=O) stretch), 1624 (C=C stretch), 1391 (C=C bending), ^1^HNMR (400 MHz, CDCl_3_, δ): 9.829 (s, 1H, OH of phenol), 7.7 (m, 1H, ArH), 7.583–7.369 (m, 5H, ArH), 7.276–7.249 (m, 1H, ArH), 7.140–7.114 (m, 1H, ArH), 6.873–6.829 (m, 1H, ArH) 6.769–6.711 (m, 1H, ArH), 3.928 (s, 3H –CH_3_) ^13^CNMR (400 MHz CDCl_3_, δ): 198.797 (C=O) 56.122 (aliphatic carbon), 190.645 (C-O), 148.941, 146.004, 144.917, 138.520, 136.966, 133.161, 132.713, 128.670, 122.864, 120.347, 119.384, 113.244, 110.669. ESI–MS: (m/z) M^+^, 254.0480.

#### (2*E*)-3-(2-aminophenyl)-1-phenylprop-2-en-1-one (11)

Yield, 39%, MP: 120–122 °C, UV–visible (EtOH) λmax: 246 (2.2355), 296 (2.0668), 362 (1.4537) IR (KBr cm^1^): 3856 (2v, (C=O), s-trans), 3715 (2ν, (C=O), s-cis), 3403 (NH-C stretch), 1858 (C=O stretch), 1622 (C=C stretch), 1213 (C-N bending). ^1^HNMR (400 MHz, CDCl_3_, δ): 8.586 (S, 2H, NH_2_), 8.133 (d, 7.2 Hz, 1H, ArH), 8.006–7.788 (m, 1H, ArH), 7.681–7.440 (m, 2H, ArH), 7.360–7.185 (m, 2H, ArH), 7.099–6.951 (m, 2H, ArH),6.901–6.855 (m, 1H, ArH), 6.756–6.624 (m, 2H, ArH), ^13^CNMR (400 MHz CDCl_3_, δ): 194.192 (C=O), 146.042, 144.965, 140.703, 137.795, 135.821, 135.297, 133.428, 130.387, 129.042, 124.809, 122.578, 120.166, 117.448, 116.409. ESI–MS (m/z): M + NH_4,_ 241.0603.

#### (2*E*)-3-(3, 4-dihydroxyphenyl)-1-phenylprop-2-en-1-one (12)

Yield: 20%, mp: 186–188 °C, UV–visible (EtOH) λmax (nm) log (ε): 246 (2.2099), 288 (2.0318), 392 (2.1181), IR (KBr cm^1^):3814 (2v, (C=O), s-trans), 3682 (2v, (C=O) s-cis), 3478 (OH stretch), 3034 (C-H stretch), 1808 C=O stretch, 1637 C=C stretch, 1427 C=C bending, ^1^HNMR (400 MHz, CDCl_3_, δ): 8.002–7.924 (m, 1H, ArH), 7.718–7.676 (m, 1H, ArH), 7.553–7.438 (m, 3H, ArH), 4.376–7.118 (m, 4H, ArH), 6.985–6.805 (m, 1H, ArH). ESI–MS (m/z): M^+^, 240.0968.

### Molecular docking studies

Molecular docking studies were carried out to have a better understanding on the synthesized compounds interaction at the molecular level with the pathogenic microbial organisms. Two Gram-positive, two Gram-negative and two fungi strains were used in the in silico evaluation of the antimicrobial activity. The Gram-negative bacteria targets used included UDP‐3‐O‐((*R*)‐3‐hydroxymyristoyl)‐*N*‐acetyl (PDB ID: 3P3E) for *Pseudomonas aeruginosa* and glutaredoxin (PDB ID: 1GRX) for *E. coli*. Gram-positive bacteria targets included: DNA gyrase (PDB ID: 3G75) for *Staphylococcus aureus* and Glucosamine-6-phosphate synthase (PDB code: **2VF5)** for *S.typhi* The fungi targets were dihydrofolate reductase (PDB ID 1AI9) for *Candida albicans*, (PDB ID: 1WS3) for *Aspergellus niger.* The 3D structures of these drug targets with their co-crystallized ligands were obtained from the Protein Data Bank (http://www.rcsb.org) with the resolution of 2.62 Å. Auto-Dock tools 1.5.4 was used to determine the grid box size for the potential binding site. The structure of the compounds was optimized with Gaussian 09^37^. The determined dimension was X = 26, Y = 26, Z = 26 with 1.00 Å as the grid spacing. Lamarckian genetic algorithm method was applied to obtain the optimum binding site for the ligand^38^. Gasteiger charges were computed using Auto-Dock tools graphical user interface supplied by MGL tools^39^. We however used optimal interactions and the best Auto-Dock socre for the interpretation of the best conformation.

#### In silico prediction

The physicochemical properties, lipophilicity, water solubility, pharmacokinetics, Druglikeness, and medicinal chemistry properties of the synthesized compounds were assessed using SwissADME online software.

## Biological studies

### Antimicrobial activity of the synthesized chemical compounds

#### Methodology

Nutrient agar and potato dextrose agar were prepared using manufacturer’s guide and sterilized by autoclaving at 121 °C for 15 min and stored for 42 °C until used.

#### Test microorganisms used

The test microorganisms used (S*taphylococcus aureus, Escherichia coli, Bacillus subtilis, Pseudomonas aeruginosa, Salmonella typhi, Candida albicans, and Aspergillus niger*) were clinical isolates obtained from the Department of Pharmaceutical Microbiology and Biotechnology Laboratory, University of Nigeria, Nsukka. The test organisms were validated using 0.5 MacFaland turbid equivalents.

#### Preparation of the different concentration of the compounds used

A 5 mg/mL stock solution of the compounds were obtained by dissolving 10 mg of the compounds in 2 mL of 50% DMSO. Different concentrations (mg/mL) of 1.0, 0.9, 0.8, 0.7, 0.6, 0.5, 0.4, 0.3, 0.2 and 0.1 were obtained using serial dilution.

*Control test (standard)* The standard antibiotic used was ofloxacin, ciprofloxacin and fluconazole.

#### Experimental

The modified methods of Cowan^34^ was adopted in the antimicrobial assay. Different concentrations of the synthesized molecules were transferred into sterilized Petri dish, and 16 mL of sterile molten agar was added and allowed to gel. Using a permanent marker, seven equal parts were made on the plates and then the test microorganisms were added on the segments, and labeled. The culture plates were incubated for 24 h at 37 °C for bacterial and 48 h at 25 °C for fungi. After incubation, the plates were observed for sensitivity and further incubated for 24 h at 37 °C, and 48 h at 25 °C to evaluate the bactericidal and fungicidal activity respectively.

### Antioxidant activity

#### Ferrous ion chelating activity

The chelating of ferrous ions by the synthesized compounds were evaluated employing the method of Singh and Rajini^40^. Different concentrations of the compounds were added to 100 μL of 2 mM ferrous sulphate solution and 300 μL of 5 mM ferrozine and mixed. The mixture was incubated at room temperature for 10 min. The absorbance of the solution was recorded at 562 nm. Ethylene diamine tetracetate (EDTA) was used as standard. The tests were carried out in triplicate and the percentages inhibition were calculated using :

$$Percentage\, of \,inhibition = \frac{{Abs_{control} - Abs_{test } }}{{Abs_{control} }} \times 100$$

## Results and discussion

### Chemistry

**Scheme 1:**
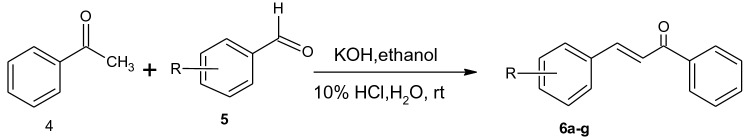
Synthetic route to new chalcone derivatives.

**Scheme 2:**
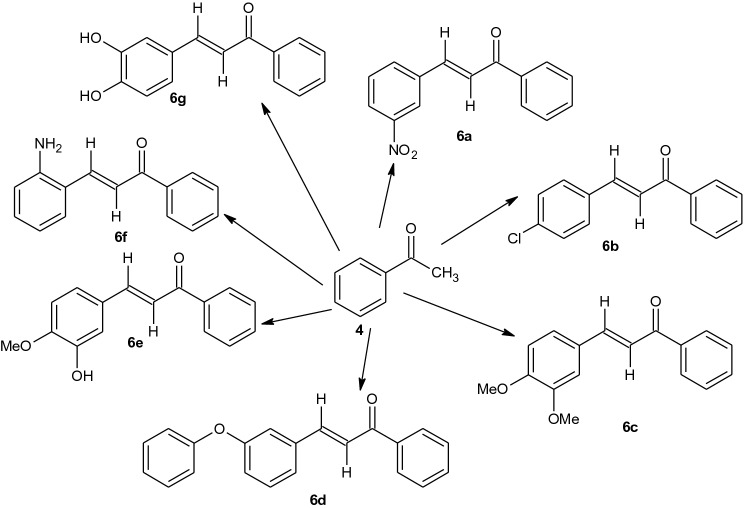
Synthesis of New chalcone derivatives.

Reaction of acetophenone (**4**) with substituted benzaldehyde (**5**) in basic medium, formed the chalcone derivatives (**6a–g,** Scheme 1) which were characterized using UV visible, FTIR, NMR, and HRMS (Scheme 2).

## Spectral characterization

In the FTIR, the band at 3167 cm^−1^ and 3478 cm^−1^ in compounds **6e** and **6g** respectively are due to OH, band at 3403 cm^−1^ in compound **6f** is due to C-NH of the amine group. The bands between 1808–1878 cm^−1^ in all the compounds are due to C=O stretch conjugated with olefinic bond while the bands at 1601–1637 cm^−1^ are due to the C=C stretch that conjugated with a carbonyl group of ketone. The band at 821 cm^−1^ in compound **6b** is due to C–Cl stretch. These bands indicates successful formation of the desired chalcones.

In the ^1^HNMR, the peak at 9.83 ppm in compound **6e** is assigned to the OH of the phenolic group. The appearance of doublet at 7.91–8.133 ppm and multiplets at 7.18–8.51 ppm were indicative of the successful formation of the desired chalcones. The appearance of singlets at 3.94 and 3.81 ppm in compound **6c** confirmed the presence of dimethyl group, while the appearance of singlet at 3.93 ppm in compound **6e** indicates the presence of a methyl group. All these indicate successful formation of the target products.

The peaks at 189–198 ppm in the ^13^CNMR indicates the presence of C=O of ketone. The peaks at 190 ppm in compounds **6c**, **6d** and **6e** are due to C–O. All the aromatic and aliphatic peaks were accounted for in the carbon-13 NMR. The carbon-13 NMR showed all the peaks expected of successful coupled products.

The high resolution mass spectrometer (HRMS) peak of the derivatives appeared as molecular ions (M^+^). The results corresponded to three decimals with the calculated values. The spectra used for the characterisation of the new compounds are available as supporting documents.

### Absorption, distribution, metabolism and excretion prediction

One of the procedures in drug development processes is the ability of the drug target to be orally bioP available^41^. Lipinski rule proposed some rules that drug target must have; the molecular weight ≤ 500, hydrogen bond acceptor (HBA) ≤ 10, hydrogen bond donor (HBD) ≤ 5, lipophilicity (logP) ≤ 5. The rule highlights feasible bioavailability problem if more than two tests are breached^42^. All the synthesized compounds obeyed the rule except compound **6d** which has logP of 5.71 as shown in Table 1.

**Table 1:** Physicochemical properties.

S/N	formula	Molar mass (g/mol)	Num. heavy atoms	Num. arom. heavy atoms	Fraction Csp3	Num. rotatable bonds	Num. H-bond acceptors	Num. H-bond donors	Molar refractivity	TPSA (Å)^2^
**6a**	C_15_H_11_NO_3_	253.25	19	12	0.00	4	3	0	75.07	62.87
**6b**	C_15_H_11_ClO	242.70	17	12	0.00	3	1	0	71.26	17.07
**6c**	C_17_H_16_O_3_	268.31	20	12	0.12	5	3	0	79.23	35.53
**6d**	C_21_H_16_O_2_	300.35	23	18	0.00	5	2	0	92.76	26.30
**6e**	C_16_H_14_O_3_	254.28	19	12	0.06	4	3	1	74.76	46.53
**6f**	C_15_H_13_NO	223.27	17	12	0.00	3	1	1	70.65	43.09
**6g**	C_15_H_12_O_3_	240.25	18	12	0.00	3	3	2	70.29	57.53

The values of the consensus Log*P*_o/w_ is an indication that the compounds are highly lipophilic and they will be distributed in the lipid regions of the body to a greater extent. The results (Table 2) indicates that the compounds will also have good absorption, distribution, metabolism and excretion characteristics since the Log*P*_o/w_ is > 2 and < 6^43^.

**Table 2:** Lipophilicity.

S/N	Log*P*_o/w_ (iLOGP)	Log*P*_o/w_ (XLOGP)	Log*P*_o/w_ (WLOGP)	Log*P*_o/w_ (MLOGP)	Log*P*_o/w_ (SILICOS-IT)	Consensus Log*P*_o/w_
**6a**	2.17	3.54	3.38	2.26	1.75	2.62
**6b**	2.82	3.71	4.13	3.96	4.57	3.84
**6c**	2.99	4.16	3.49	2.66	4.01	3.46
**6d**	3.48	4.61	5.27	4.24	5.10	4.54
**6e**	2.17	3.31	3.19	2.42	3.48	3.02
**6f**	2.31	3.01	3.06	2.78	3.21	2.88
**6g**	1.98	3.44	2.89	2.17	2.96	2.69

The water solubility of the compounds (Table 3) as predicted by SwissADME software shows that compound **6e, 6f** and **6g** will have more absorption and distribution in the aqueous region of the body than compound **6a, 6b** and **6c** while compound **6d** will have poor absorption and distribution in the aqueous region of the body.

**Table 3:** Water solubility.

S/N	ESOL			ALI			SILICOS-IT		
	Log S	Solubility	Class	Log S	Solubility	Class	Log S	Solubility	Class
**6a**	− 3.84	3.63e−02 mg/ml; 1.43e−04 mol/l	S	− 4.55	7.21e−03 mg/ml; 2.85e−05 mol/l	MS	− 4.34	1.16e−02 mg/ml; 4.59e−05 mol/l	MS
**6b**	− 4.01	2.39e−02 mg/ml; 9.85e−05 mol/l	MS	− 3.76	4.22e−02 mg/ml; 1.74e−04 mol/l	S	− 5.58	6.46e−04 mg/ml; 2.66e−06 mol/l	MS
**6c**	− 4.24	1.55e−02 mg/ml; 5.78e−05 mol/l	MS	− 4.61	6.52e−03 mg/ml; 2.43e−05 mol/l	MS	− 5.21	1.66e−03 mg/ml; 6.18e−06 mol/l	MS
**6d**	− 4.86	4.19e−03 mg/ml; 1.39e−05 mol/l	MS	− 4.89	3.89e−03 mg/ml; 1.30e−05 mol/l	MS	− 7.20	1.90e−05 mg/ml; 6.32e−08 mol/l	PS
**6e**	− 3.71	5.01e−02 mg/ml; 1.97e−04 mol/l	S	− 3.96	2.77e−02 mg/ml; 1.09e−04 mol/l	S	− 4.51	7.86e−03 mg/ml; 3.09e−05 mol/l	MS
**6f**	− 3.44	8.02e−02 mg/ml; 3.59e−04 mol/l	S	− 3.58	5.88e−02 mg/ml; 2.63e−04 mol/l	S	− 4.6	5.60e−03 mg/ml; 2.51e−05 mol/l	MS
**6g**	− 3.79	3.88e−02 mg/ml; 1.61e−04 mol/l	S	− 4.33	1.13e−02 mg/ml; 4.69e−05 mol/l	MS	− 3.81	3.72e−02 mg/ml; 1.55e−04 mol/l	S

*S *soluble, *MS* moderately soluble, *PS* poorly soluble.

The results of pharmacokinetics prediction is presented in Table 4. The high GI adsorption is an indication that the compounds will be broken down and digested very quickly in the body and it will be largely absorbed by the small intestine. The BBB (blood brain barrier) regulates an external surface interaction between the blood and the brain, the BBB result above, it shows that the compounds can have access to the central nervous system (CNS), so it can be used to treat any infection of the central nervous system. The negative value of Log K_p_ shows that it is likely to have a low skin permeability.

**Table 4:** Pharmacokinetics.

S/N	GI absorption	BBB permeant	P-gp substrate	CYP1A2 inhibitor	CYP2C19 inhibitor	CYP2C9 inhibitor	CYP2D6 inhibitor	CYP3A4 inhibitor	Log K_p_ (cm/s)
6a	High	Yes	No	Yes	Yes	Yes	No	No	− 5.33
6b	High	Yes	No	Yes	Yes	Yes	No	No	− 5.15
6c	High	Yes	No	Yes	Yes	Yes	Yes	No	− 4.98
6d	High	Yes	No	Yes	Yes	Yes	No	Yes	− 4.86
6e	High	Yes	No	Yes	Yes	Yes	No	Yes	− 5.50
6f	High	Yes	No	Yes	Yes	Yes	No	No	− 5.52
6g	High	Yes	No	Yes	No	Yes	No	Yes	− 5.32

Permeability glycoprotein (P-gp) major role is to protect the central nervous system from xenobioties. The synthesized compound is not a good P-gp substrate and so it is not a good inhibitor of xenobiotics. The drug is an inhibitor of CPY450 enzymes, so it blocks the metabolic activities of one or more CYP450 enzymes.

The compounds obeyed Lipinski, Ghose, Veber, Egan and muegge rule of five (Table 5) therefore, they will be orally bioactive in systematic circulation and the bioavailability score of 0.55 shows that the compounds can act as good oral drugs.

**Table 5:** Druglikeness.

S/N	Lipinski	Ghose	Veber	Egan	Muegge	Bioavailability score
6a	Yes	Yes	Yes	Yes	Yes	0.55
6b	Yes	Yes	Yes	Yes	No	0.55
6c	Yes	Yes	Yes	Yes	Yes	0.55
6d	Yes	Yes	Yes	Yes	Yes	0.55
6e	Yes	Yes	Yes	Yes	Yes	0.55
6f	Yes	Yes	Yes	Yes	Yes	0.55
6g	Yes	Yes	Yes	Yes	Yes	0.55

The pan-assay interference compounds (PAINS) and Brenk alert allows the identification of potentially problematic fragments in the studied molecules, from the Table 6, the compounds are said to contain problematic fragments. Compound **6e** has leadlikeness which implies that the compound can be subjected to chemical modifications while compounds **6a, 6b, 6c, 6d, 6f** and **6g** has no leadlikeness therefore, they cannot be subjected to chemical modifications^44^. The synthetic accessibility score for the compounds are far less than 5 therefore, the compounds can easily be synthesized.

**Table 6:** Medicinal chemistry.

S/N	PAINS	Brenk	Leadlikeness	Synthetic accessibility
6a	0 alert	3 alert	No	2.65
6b	0 alert	1 alert	No	2.42
6c	0 alert	1 alert	No	2.61
6d	0 alert	1 alert	No	2.84
6e	0 alert	1 alert	Yes	2.48
6f	0 alert	2 alert	No	2.53
6g	1 alert	2 alert	No	2.42

**Figure 1:**
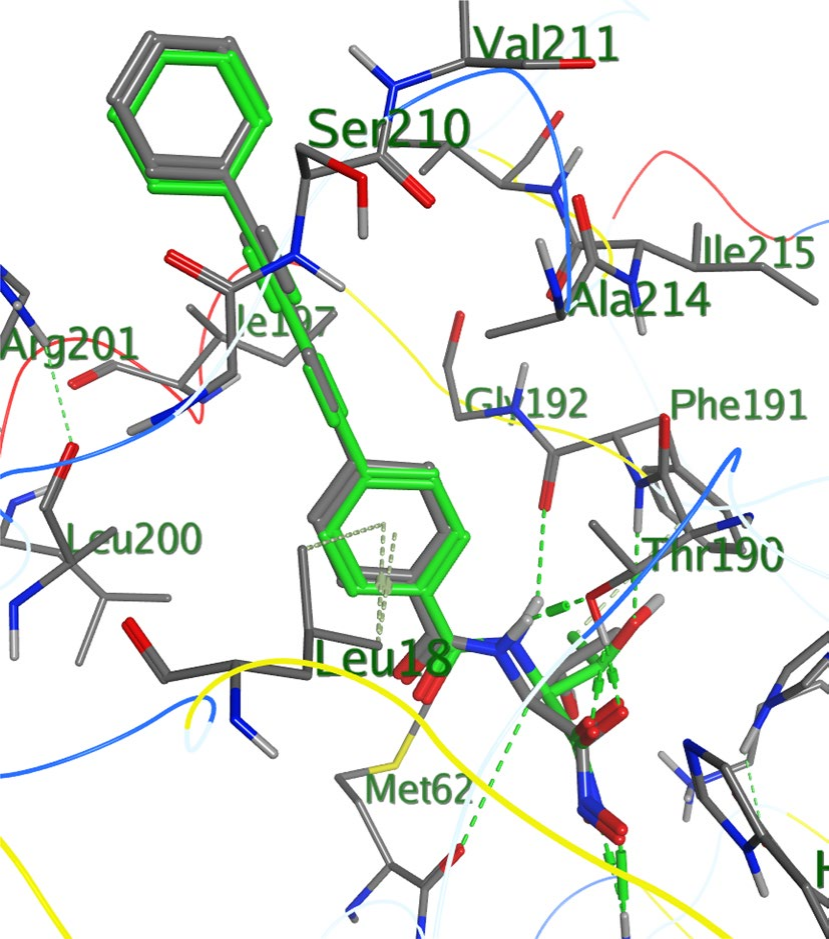
Validation of docking protocol using 3P3E (retrieved co-crystallized ligand (green) is docked into the binding cavity of 3P3E and is superimposed on the co-cystallized ligand (grey) intact with 3P3E.

Looking at their binding affinities compared with ciprofloxacin and fluconazole as the standards, the compound all had good binding affinity with the target microorganism proteins but, compounds **6e** and **6g** showed better binding affinity when compared with the standard as shown in Table 7. The docking protocol was validated using 3P3E as shown in Fig. 1.

**Table 7:** Binding free energy (ΔG) of the compounds.

	Gram-positive bacteria		Gram-negative bacteria		Fungi	
	*B. subtilis * (2V F5)	*S.aureus* (3G75)	*P.aeruginosa * (3P3E)	*E.coli* (1GRX)	*C.albicans * (1AI9)	*A.niger* (1WS3)
**Compound**						
**6a**	− 10.29	− 10.86	− 10.49	− 9.20	− 10.91	− 9.46
**6b**	− 9.32	− 9.40	− 9.49	− 8.07	− 10.17	− 9.44
**6c**	− 10.69	− 10.55	− 11.45	− 8.99	− 11.70	− 9.58
**6d**	− 9.20	− 10.81	− 10.48	− 8.79	− 11.68	− 9.60
**6e**	− 11.54	− 11.32	− 13.09	− 10.15	− 11.47	− 10.95
**6f**	− 8.97	− 9.81	− 9.61	− 8.46	− 11.00	− 9.07
**6g**	− 12.53	− 11.99	− 12.54	− 10.85	− 12.39	− 10.77
Std drug	− 12.93	− 12.12	− 13.22	− 11.20	− 10.70	− 9.40
Native ligand	− 13.61	− 10.29	− 11.68	− 12.81	− 19.04	− 7.03

Standard drugs: ciproflocin and fluconazole.

**Figure 2:**
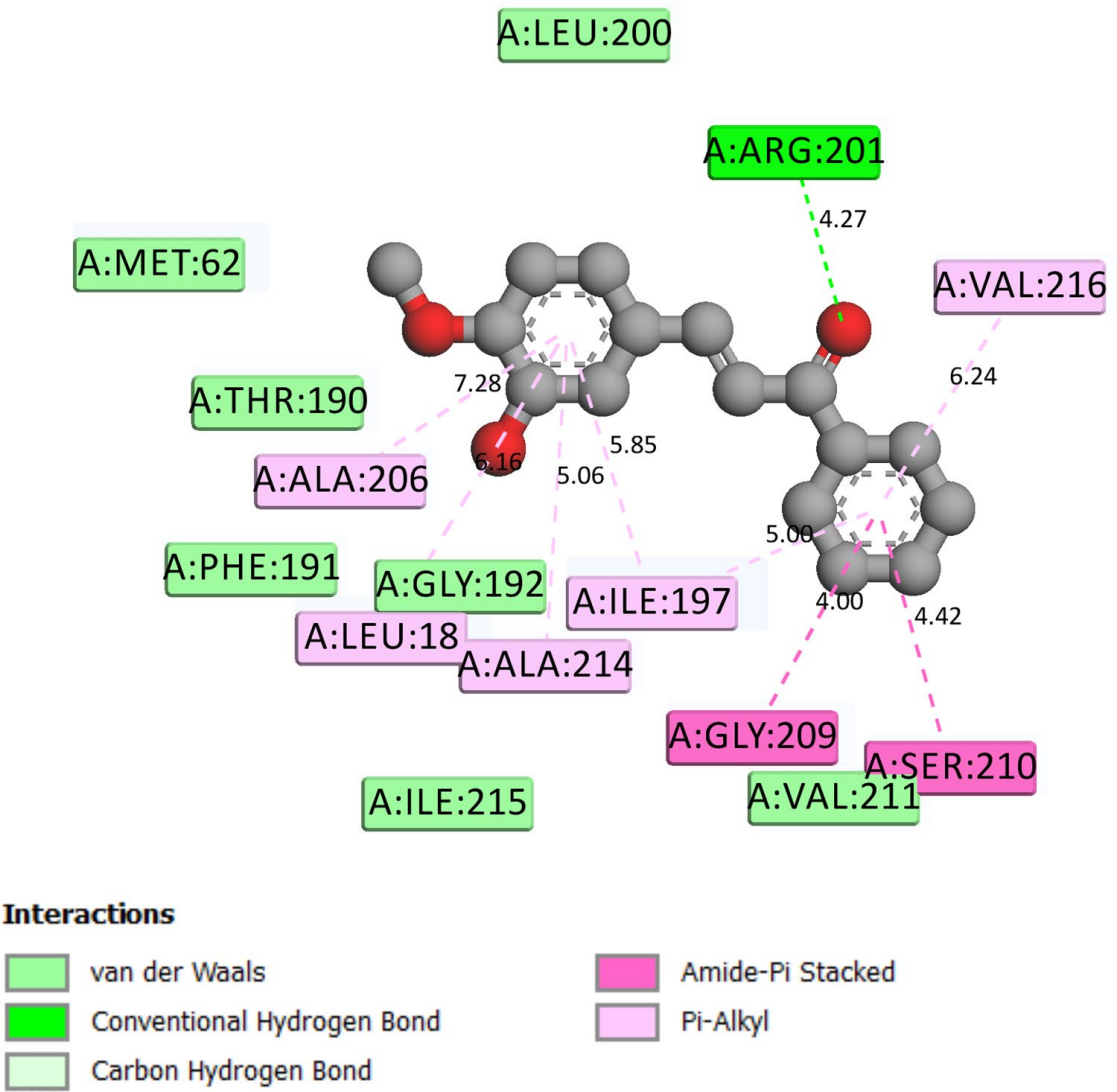
Rrepresentation of the binding interactions between compound **6e** and the amino residues of 3P3E.

**Figure 3:**
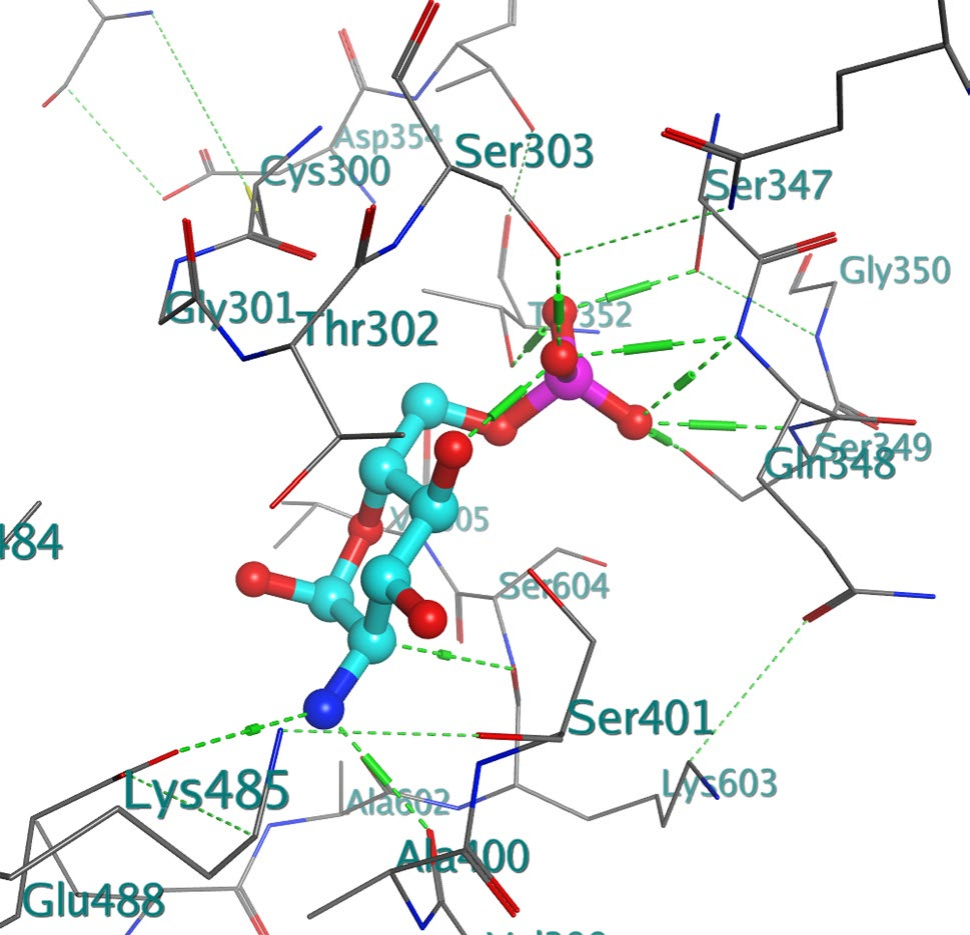
Binding pose of compound **6g** in the binding cavity of 2VF5.

**Figure 4:**
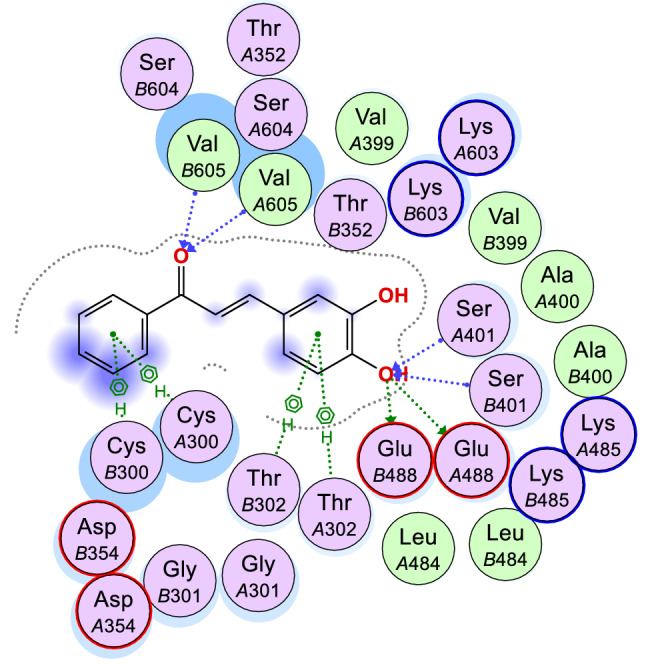
2D representation of the binding interactions between compound **6g** and the amino residues of 2VF5.

**Figure 5:**
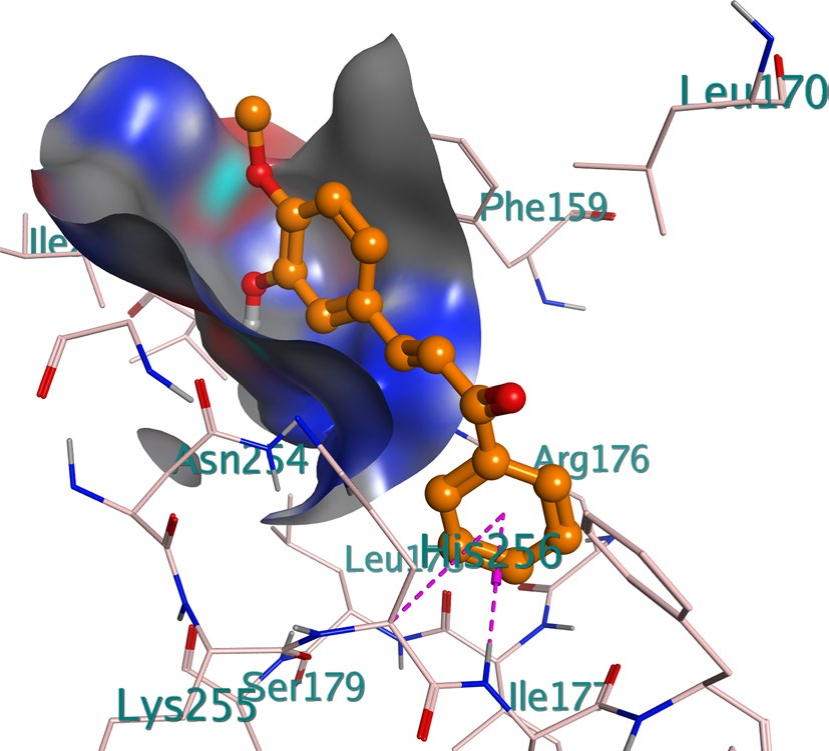
Binding pose of compound 6e in the binding cavity of 1WS3.

**Figure 6:**
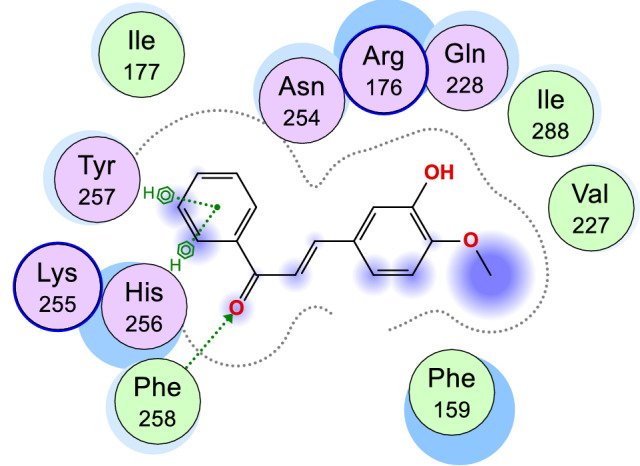
2D representation of the binding interactions between compound 6e and the amino residues of 2VF5.

Closer studies on compounds **6e** and **6g** were carried out with the receptor 2VF5 and 1WS3 as shown in Figs. 2, 3, 4, 5 and 6 to gain more insight about the compound interaction with the proteins of the microorganisms. Figure 2 showed the binding interaction between compound **6e** with the amino acid 3P3E of the receptor respectively. Hydrogen bond; ARG:201, Van der waals; MET: 62, THR: 190, PHE:191, GLY:192, ILE:215, VAL:211, amide-Pi stack; GLY:209, SER:210 while Pi-alkyl; ALA: 206, LEU:18, ALA:214 and ILE:197. These representations showed that there are significant hydrogen bond interaction between the amino residues 2VF5 of the organisms and the compound as indicated on the Fig. 6 and Table 8. Figure 3 showed the binding pose of compound **6g** in the cavity of 2VF5, an indication of outstanding hydrogen bonding interaction between the compound and the amino residues making the compound to have high binding affinity with the organisms. Table 9 also showed the interaction between the amino residue and the compound **6g**; the proteins involved the types of hydrogen interactions, bond lengths and bonding energies. Because of these interactions, compounds **6e** and **6g** can be said to have drug target since they show good antagonist on the biochemical processes of the receptors. We observed that the standard drugs and co-crystalized ligands had better antibacterial activity than the reported derivatives, however, the synthesized derivatives showed better antifungal activity in the in silico experiment.

**Table 8:** Chemical interactions of compound 6g with amino residues of 2VF5.

Ligand	Receptor	Interaction	Distance (Å)	E (kcal/mol)
O 1	OE2 GLU 488	H-donor	2.96	− 0.7
O 1	OE2 GLU 488	H-donor	2.96	− 0.7
O 1	N SER 401	H-acceptor	2.98	− 0.3
O 1	N SER 401	H-acceptor	2.98	− 0.3
O 12	N VAL 605	H-acceptor	3.36	− 0.5
O 12	N VAL 605	H-acceptor	3.36	− 0.5
6-ring	CB CYS 300	pi-H	4.16	− 0.2
6-ring	N THR 302	pi-H	4.46	− 0.3
6-ring	CB CYS 300	pi-H	4.16	− 0.2
6-ring	N THR 302	pi-H	4.46	− 0.3

**Table 9:** Chemical interactions of compound 6e with amino residues of 2VF5.

Ligand	Receptor	Interaction	Distance (Å)	E (kcal/mol)
O 13	CE1 PHE 258	H-acceptor	3.82	− 0.2
6-ring	CA HIS 256	pi-H	4.26	− 0.5
6-ring	N TYR 257	pi-H	4.06	− 1.3

### In vitro antimicrobial activities

The results of the antimicrobial assays (Tables 10 and 11), revealed that all the compounds were active against S*taphylococcus aureus and Bacillus subtilis* with minimium inhibitory concentrations (MIC) between 0.4–0.6 mg/mL. Compounds **6a**, **6c** and **6d** have moderate activities on *Salmonella typhi.* Compounds **6b** and **6c** have moderate activity on *Escherichia coli.* Compound 109 has moderate activity on *Aspergillus niger*, compounds **6a** and **6e** had poor activities on *Escherichia coli and Aspergillus niger* while compound **6c** had moderate inhibitory activity on *Aspergillus niger*, compound **6e** had moderate inhibition on *Pseudomonas aeruginosa.* All the compounds except compound **6e** had no inhibition activity against *Pseudomonas aeruginosa.* Only compound **6d** had activity on *Pseudomonas aeruginosa.* All the compounds screened had poor activity on *Aspergillus niger* except compound **6c** that showed moderate activity. None of the reported compounds had antimicrobial activity comparable with the standard drugs. We therefore would consider further optimization of the active derivatives. We also noticed that in spite of the good in silico results against C. albicans protein, the compounds were inactive against C. albicans suggesting that the concentration used for the assay is below the active concentration. However, the in silico results is in agreement with the results obtained from the invitro experiment which strongly suggest the inhibition of the tested enzymes in the in vitro experiments.

**Table 10:** Minimum inhibitory concentration (MIC, mg/mL).

Compound	*E. coli*	*S. typhi*	*S. aureus*	*B. subtilis*	*P. aeruginosa*	*C. albicans*	*A. niger*
**6a**	0.9	0.7	0.4	0.6	NA	NA	0.9
**6b**	0.6	0.8	0.6	0.5	NA	NA	0.9
**6c**	0.7	0.7	0.4	0.4	NA	NA	0.9
**6d**	NA	0.6	0.4	0.5	NA	0.8	0.9
**6e**	0.9	0.8	0.5	0.4	0.8	NA	0.9
**6f**							
**6g**							
Ciprofloxacin	0.02	0.015	0.025	0.020	0.025	NA	NA
Fluconazole	NA	NA	NA	NA	NA	0.020	0.005

*NA* no activity.

**Table 11:** Ferrous ion chelating activity.

% of inhibition					
Compound	Concentration (µg/ml)				
	125	250	500	1000	IC 50 (µg/ml)
**6a**	NI	43.75	85.94	94.79	246.5
**6b**	NI	NI	NI	NI	0.0
**6c**	81.77	89.58	86.46	93.75	1.71
**6d**	NI	NI	70.31	70.31	353.15
**6e**	70.31	73.43	73.44	75.52	6.30
**6f**	67.71	75.52	67.71	80.73	2.33
EDTA	54.17	54.17	55.21	71.35	3.04

*NI *no inhibition.

### Antioxidant activity

The compounds were assessed for their *in-vitro* antioxidant activities using ethylenediamine tetracetate (EDTA) as the standard. Compounds **6c**, **6e** and **6g** gave excellent inhibitory activities above those of the standard. Compound **6a** gave good activity at 500 μg/mL and 100 μg/mL concentrations but, below the standard at 250 μg/ml and no inhibition at 125 μg/mL. Compound **6d** had good inhibition at 500 μg/mL and 1000 μg/mL but, no inhibition at 125 μg/mL and 250 μg/mL. Compound **6b** was found to be inactive in all the concentrations. The IC50 values showed that only compounds **6c** and **6f** had better antioxidant activity.

## Conclusions

In this paper, we have described a versatile approach to obtain chalcone derivatives. All the compounds were evaluated for their antimicrobial and antioxidant activities. Compound **6b** was the most active against *E. Coli,* compound **6d** was the most potent against *S. typhi,* compounds **6a**, **6c** and **6d** had the same activity (MIC 0.4 mg/mL) againt *S. aureus,* compounds **6c** and **6e** were the most active (MIC 0.4 mg/mL) against *B. subtilis* only compound **6d** showed activity (MIC 0.8 mg/mL) against *C. albicans*, only compound **6e** showed activity (MIC 0.8 mg/mL) against *P. aeruginosa* while all the compounds were active against *A*. *niger*. Compound **6c** had highest antioxidant activity. SwissADME was used to predict the absorption, distribution, metabolism and excretion properties of the compounds and the results showed that the reported derivatives have druggable properties. In particular, compound 6e was reported to be drug-like.
